# Analyzing AbrB-Knockout Effects through Genome and Transcriptome Sequencing of *Bacillus licheniformis* DW2

**DOI:** 10.3389/fmicb.2018.00307

**Published:** 2018-02-26

**Authors:** Cheng-Cheng Shu, Dong Wang, Jing Guo, Jia-Ming Song, Shou-Wen Chen, Ling-Ling Chen, Jun-Xiang Gao

**Affiliations:** ^1^Agricultural Bioinformatics Key Laboratory of Hubei Province, College of Informatics, Huazhong Agricultural University, Wuhan, China; ^2^State Key Laboratory of Agricultural Microbiology, College of Life Science and Technology, Huazhong Agricultural University, Wuhan, China

**Keywords:** *Bacillus licheniformis*, reference genome, transcriptome, AbrB-knockout, differentially expressed genes, bacitracin biosynthesis

## Abstract

As an industrial bacterium, *Bacillus licheniformis* DW2 produces bacitracin which is an important antibiotic for many pathogenic microorganisms. Our previous study showed AbrB-knockout could significantly increase the production of bacitracin. Accordingly, it was meaningful to understand its genome features, expression differences between wild and AbrB-knockout (ΔAbrB) strains, and the regulation of bacitracin biosynthesis. Here, we sequenced, *de novo* assembled and annotated its genome, and also sequenced the transcriptomes in three growth phases. The genome of DW2 contained a DNA molecule of 4,468,952 bp with 45.93% GC content and 4,717 protein coding genes. The transcriptome reads were mapped to the assembled genome, and obtained 4,102∼4,536 expressed genes from different samples. We investigated transcription changes in *B. licheniformis* DW2 and showed that ΔAbrB caused hundreds of genes up-regulation and down-regulation in different growth phases. We identified a complete bacitracin synthetase gene cluster, including the location and length of *bacABC, bcrABC*, and *bacT*, as well as their arrangement. The gene cluster *bcrABC* were significantly up-regulated in ΔAbrB strain, which supported the hypothesis in previous study of *bcrABC* transporting bacitracin out of the cell to avoid self-intoxication, and was consistent with the previous experimental result that ΔAbrB could yield more bacitracin. This study provided a high quality reference genome for *B. licheniformis* DW2, and the transcriptome data depicted global alterations across two strains and three phases offered an understanding of AbrB regulation and bacitracin biosynthesis through gene expression.

## Introduction

*Bacillus licheniformis* (*B. licheniformis*) is a Gram-positive bacterium that is widely used in multiple fields: in agriculture as a probiotic and microbial fertilizer (Pötter et al., 2001), and in biotechnology industry for the production of enzymes, acetoin and poly-γ-glutamic acid (γ-PGA) (Veith et al., 2004; Liu et al., 2011; Konglom et al., 2012). This facultative anaerobic organism can also produce a variety of antibiotics such as bacitracin, which is an important peptide antibiotic for many pathogenic microorganisms secreted by certain strains of *B. licheniformis* and *B. subtilis* (Johnson et al., 1945; Mcinerney et al., 1990). The branched cyclic dodecyl peptide bacitracin is the most active against other Gram-positive and certain Gram-negative microorganisms, which is synthesized by a large non-ribosomal multi-enzyme complex *bacABC* (Konz et al., 1997). The AbrB gene of *B. licheniformis* and *B. subtilis* is known as an important global transcription repressor, but also act as an activator for some genes by binding to promoters (Kim et al., 2003). AbrB has a wide range of regulatory functions, including cell wall and membrane synthesis, bacterial chemotaxis, antibiotic synthesis, amino acid synthesis and transport, protein modification and so on (Qian et al., 2002; Strauch et al., 2007). AbrB directly regulates more than 100 genes and indirectly influence hundreds genes. The evidence suggests that expression level of AbrB is inhibited by another regulatory gene Spo0A-P, leading to an opposite tendency of AbrB gene expression compares with that of Spo0A-P, i.e., high expression during the logarithmic phase and low expression during stationary phase (Strauch et al., 1990; Shafikhani and Leighton, 2004; Banse et al., 2008; Chumsakul et al., 2011).

Since the project of *B. subtilis* genome was completed as the first gram-positive bacterium (Kunst et al., 1997), more and more *Bacillus* strains have been sequenced including *B. licheniformis* ATCC14580, 9945A, WX-02 and DSM13; *B. subtilis* CGMCC 12426, BSD-2, and so on (Veith et al., 2004; Guo et al., 2015; Li et al., 2015; Liu et al., 2016). In contrast, some other studies are limited to single or several proteins and metabolites, for example, the production of increased isopentenyl pyrophosphate (IPP) (Cain et al., 1993; Chalker et al., 2000), the secretion of exopolysaccharides (Pollock et al., 1994) and *bcrABC* protein transporting bacitracin in *B. subtilis* (Podlesek et al., 1995, 2000). Although these studies provide great advances, there has never been a comprehensive research focused on the effects of AbrB-knockout in term of whole genome and transcriptome, especially the effect on the bacitracin gene cluster.

Our previous study showed that AbrB-knockout could significantly increase the production of bacitracin, and the yields were 17.5% higher than that of the wild-type strain (Wang et al., 2017). As an industrial *B. licheniformis* strain, DW2 can produce an extra yield using the improvement way of AbrB-knockout in industrial application. In the present study, we sequenced, annotated the genome and transcriptome of an important industrial *B. licheniformis* strain DW2, and obtained the following results. (1) The genome of *B. licheniformis* DW2 is 4,468,952 bp with 45.93% GC content and 4,717 predicted coding sequences (CDSs). (2) *B. licheniformis* DW2 has good collinearity with other *B. licheniformis* strains, and a unique ∼100 kb genomic sequences. (3) N-6-methylated adenines (6mA) and 4-methylated cytosines (4mC) methylation of *B. licheniformis* DW2 was analyzed, and two 6mA motifs were identified. (4) There are 369∼1,517 differentially expressed genes (DEGs) between the transcriptome of wild and ΔAbrB strains in logarithmic growth phase, transitional phase and stationary growth phase. (5) We identified the location and length of bacitracin synthetase gene cluster in *B. licheniformis* DW2. In this gene cluster, the transporter genes *bcrABC* were significantly highly expressed in ΔAbrB strain, which supported the assumption that *bcrABC* genes could pump bacitracin out to avoid self-intoxication. The study produced a reference genome of *B. licheniformis*, also enabled us to highlight global changes in gene expression and provided theory and data support for industrial process of bacitracin production.

## Materials and Methods

### Genomic DNA Preparation

The DW2 strain used in this study was provided by China Center for Type Culture Collection (CCTCC), whose number was CCTCC M2011344. *B. licheniformis* DW2 was grown on Luria Bertani (LB) agar plates or in LB broth at 37°C, supplemented with antibiotics (tetracycline, 20 mg/mL; ampicillin, 50 mg/mL; kanamycin, 20 mg/mL) when necessary. The seed culture (1 mL) was inoculated into a 250 mL Erlenmeyer flask containing 20 mL bacitracin fermentation medium [6% soybean meal, 4.5% starch, 0.1% (NH_4_)_2_SO_4_, 0.6% CaCO_3_, pH 6.5∼7.0], followed by 40 h incubation on a rotary shaker (180 rpm) at 37°C. Genomic DNA was extracted using a standard SDS-Phenol procedure (Sambrook et al., 1982) according to the manufacturer’s instructions, combined with phenol-chloroform extraction and RNase A treatment.

### PacBio SMRT Sequencing

We determined the complete genome sequence of *B. licheniformis* DW2 using PacBio single molecule real-time (SMRT) technology. We prepared a 3 to 20 kb genomic DNA library suitable for P6/C4 chemistry. Using two SMRT cell on PacBio RSII sequencing platform, 116,486 polymerase reads with mean read length of 4,268 bp were obtained. The polymerase reads were assembled *de novo* with Hierarchical Genome Assembly Process 3 (HGAP3) within the SMRT Analysis version 2.3.0 software (Chin et al., 2013). Subsequently, the best assembly was selected, and Minimus 2 was used for trimming the circular contig (Sommer et al., 2007). The replication origin was determined by aligning the DW2 genomic sequence with *B. licheniformis* DSM13.

For identification of methylated bases and modification motifs, the “RS_Modification_and_Motif_Analysis.1” protocol in SMRT Portal under default parameter settings was used, with the assembled genome. Putative restriction modification systems have been identified using the Restriction-ModificationFinder-1.0 server^1^ based on the Restriction Enzyme database (REBASE) (Roberts et al., 2015).

### The Annotation of *B. licheniformis* DW2 Genome

After the complete sequence was obtained, the genome was annotated using the rapid annotation tool RAST (Ogata et al., 1999; Aziz et al., 2008). In addition, the unigenes were annotated by the public protein databases of Kyoto Encyclopedia of Genes and Genomes (KEGG) (Galperin et al., 2015), Cluster of Orthologous Groups of Proteins (COG) (Gene Ontology Consortium, 2015), Swiss-Prot protein^2^, NCBI non-redundant (NR^3^), and Gene Ontology (GO) (Lagesen et al., 2007) using BLASTP to get corresponding functional annotation information. As each sequence had many alignments, we retain the optimal comparison results as the annotation of the gene to ensure its accuracy. The predictions of rRNA and tRNA were performed using RNAmmer (Lowe and Eddy, 1997) and tRNAscan-SE (Zhang et al., 2000), respectively. Insertion sequence (IS) elements were predicted with IS Finder (Benson, 1999).

### Whole-Genome Alignment and Identification of Operon Structures

We constructed and visualized the multiple genome alignment of four complete genomes of *B. licheniformis* strains DW2, ATCC14580, 9945A and WX-02 using Mauve v2.4.0 (Darling et al., 2010). Pairwise collinear comparisons of the four genome sequences were performed using Mummer3 (Kurtz et al., 2004). Operon of *B. licheniformis* DW2 was identified using DOOR 2.0 algorithm for prokaryotic operon analysis (Mao et al., 2014).

### RNA Isolation and Library Preparation for ssRNA-seq

RNA samples were isolated at three time points for two bacterial samples (wild strain and ΔAbrB strain): 14 h (logarithmic growth phase), 22 h (transitional phase) and 25 h (stationary phase). Cells were collected by centrifugation at 12,000 rpm for 5 min, and then transferred to a 10-ml centrifuge tube after grinding in liquid nitrogen. Cells were then lysed in 1 mL of TRIzol for 30–60 s. To the lysate, 200 μL of chloroform was added, and the sample was then mixed by inversion and incubated at room temperature for 15 min. The sample was then centrifuged at 12,000 rpm for 15 min at 4°C, and supernatant was precipitated with an equal volume of isopropanol at room temperature for 10 min. After centrifugation, the supernatant was discarded, and the pellet was air dried and dissolved in 20–40 μL of RNase-free water. Total RNA was treated with RNase-free DNase I for 30 min at 37°C to remove genomic DNA. RNA concentration was measured using Qubit^®^ RNA Assay Kit in Qubit^®^ 2.0 Fluorometer (Life Technologies, Carlsbad, CA, United States). At the same time, RNA integrity was assessed using the RNA Nano 6000 Assay Kit of the Bioanalyzer 2100 system (Agilent Technologies, Santa Clara, CA, United States). Each sample had three replicates, and all biological replicates were processed in separate batches from each other.

Libraries for ssRNA sequencing (Novogene Experimental Department) were constructed at three time points (14, 22, and 25 h). A total amount of 3 μg RNA per sample was used as input material for the RNA sample preparations. Sequencing libraries were generated using NEBNext^®^ Ultra^TM^ Directional RNA Library Prep Kit for Illumina^®^ (NEB, United States) following manufacturer’s recommendation and index codes were added to attribute sequences to each sample. Briefly, (epicentre Ribo-Zero^TM^) fragmentation was carried out using divalent cations under elevated temperature in NEBNext First Strand Synthesis Reaction Buffer (5X). First strand cDNA was synthesized using random hexamer primer and M-MuLV Reverse Transcriptase (RNaseH-). Second strand cDNA synthesis was subsequently performed using DNA Polymerase I and RNase H. In the reaction buffer, dNTPs with dTTP were replaced by dUTP. Remaining overhangs were converted into blunt ends via exonuclease/polymerase activities. After adenylation of 3′ ends of DNA fragments, NEBNext Adaptor with hairpin loop structure were ligated to prepare for hybridization. In order to select cDNA fragments of preferentially 150∼200 bp in length, the library fragments were purified with AMPure XP system (Beckman Coulter, Beverly, MA, United States). Then 3 μl USER Enzyme (NEB, United States) was used with size-selected, adaptor-ligated cDNA at 37°C for 15 min followed by 5 min at 95°C before PCR. Then PCR was performed with Phusion High-Fidelity DNA polymerase, Universal PCR primers and Index (X) Primer. At last, products were purified (AMPure XP system) and library quality was assessed on the Agilent Bioanalyzer 2100 system.

### Mapping Reads to *B. licheniformis* DW2 Genome and Analyzing Differentially Expressed Genes

All the raw reads were initially processed to obtain clean reads by the following steps: (1) reads with adaptor were discarded; (2) reads with ambiguous bases (undetermined bases, N) larger than 10% were removed; (3) low-quality reads that contained more than 50% *Q*-value < 5 bases were discarded. After filtering, all clean reads were aligned to the assembled genome using Bowtie2 (Langmead and Salzberg, 2012), and reads with ambiguous alignments or more than three mismatches were discarded. Only uniquely mapped reads were used for further analysis. The per-base–format coverage depth and read counts were calculated using BEDTools (Quinlan and Hall, 2010). Because different samples may have different total read counts, sequencing depth, and biases, the normalized transcription level of genes was expressed in reads per kilobase of ORF per million mapped reads (RPKM) (Mortazavi et al., 2008). The transcriptome raw reads have been deposited in NCBI Sequence Read Archive database under accession number SRP045205. In addition, we generated a distance matrix for expression data based on RPKM values by using Pearson correlation as dissimilarity metric (El-Sharnouby et al., 2013). Clustering was performed and deprograms were generated using the cluster package in R.

Differentially expressed genes between wild strain and ΔAbrB strain were identified using the DESeq R package with the MARS (MA-plot-based method with Random Sampling) model (Anders and Huber, 2010). The DEGs were screened with the false discovery rate (FDR) threshold of ≤0.01 and an absolute value of fold change (FC) ≥2.

## Results and Discussion

### Genomic Features and Comparative Genomics of *B. licheniformis* DW2

**FIGURE 1:**
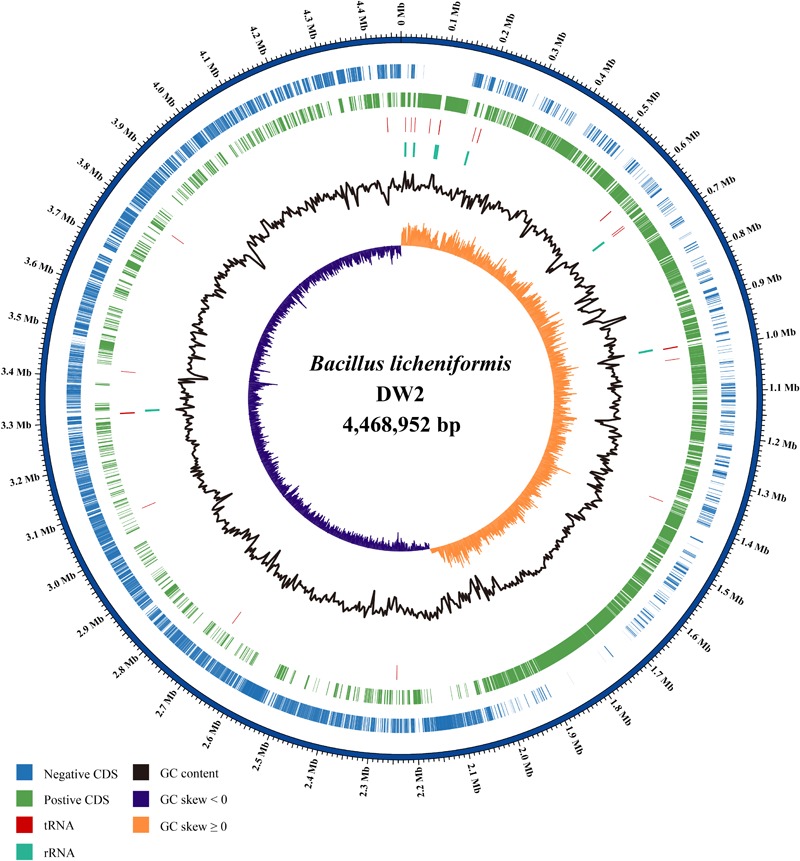
The circular plot of *B. licheniformis* DW2 chromosome. Circles are numbered from 1 (outermost) to 7 (innermost). Circle 1 represents the whole chromosome; Circles 2 and 3 show the locations of predicted CDSs on the positive and negative strands, respectively; Circle 4, tRNA genes; Circle 5, rRNA genes; Circle 6, %G + C; Circle 7, GC skew ((G - C)/(G + C)).

*Bacillus licheniformis* DW2 genome has one circular DNA with 4,468,952 bp length and 45.93% GC content. The DNA molecule (**Figure 1**) contained 4,717 predicted CDSs accounting for ∼87% of the genome. Among the predicted CDSs, 3,896 were assigned biological functions or putative functions and 821 could be annotated as hypothetical protein. In addition, 24 rRNA genes and 81 tRNA-coding genes were identified, and 71 IS elements were found. Additional information about the genome statistics is shown in **Table 1**.

**Table 1:** Genome statistics of *B. licheniformis* DW2.

Feature	Value
Genome size (bp)	4,468,952
DNA coding (bp)	3,918,654
DNA G+C (bp)	2,052,745
DNA scaffolds	1
Total genes	4,822
Protein coding genes	4,717
RNA genes	105
Genes with function predictions	3,896
Genes assigned to COGs	3,205

**FIGURE 2:**
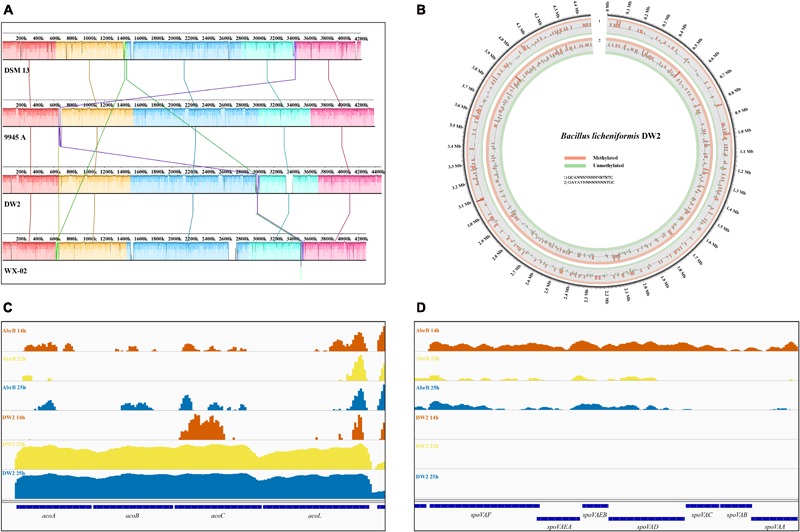
Comparative genomics of four *B. licheniformis* strains and other genomic features of *B. licheniformis* DW2. **(A)** Multiple genomic alignments of four *B. licheniformis* strains. **(B)** Distribution of 6mA motifs in *B. licheniformis* DW2 genome. **(C)** The operon acoABCL associated with acetoin catabolism in *B. licheniformis* DW2. **(D)** The stage V sporulation protein in *B. licheniformis* DW2.

To obtain further insights into *B. licheniformis* DW2 genome, we performed comparative genomics analysis with other three available *B. licheniformis* genomes: DW2, ATCC14580, 9945A and WX-02 (**Table 2**). *B. licheniformis* DW2 had the largest genome size and number of predicted CDSs, IS and tandem repeat sequence. However, the number of rRNAs and tRNAs was consistent with the others and the number of rRNAs was almost same. The multi-genome alignments showed the four genomes shared homologous blocks in order (**Figure 2A**). We found that *B. licheniformis* DW2 genome contained a specific fragment at 3.3∼3.4 Mb with 113 CDSs, most of them were hypothetical proteins and phage-associated proteins (such as phage-like protein, phage protein, phage capsid, and scaffold). Considering that only *B. licheniformis* DW2 can highly yield peptide antibiotics in the above four strains, we hypothesized that this region might be associated with the yield of peptide antibiotics and inhibition of pathogens. The *B. licheniformis* DW2 genome was collinear with the other three *B. licheniformis* strains (Supplementary Figure S1), and it has the best consistency with *B. licheniformis* 9945A.

**Table 2:** General genome features of the four *B. licheniformis* strains.

Strain	Size (Mp)	GC%	No. CDS	No. rRNA	No. tRNA	No. Insertion sequence	No. Tandem repeats
*B. licheniformis* DW2	4.47	45.93	4,717	24	81	71	144
*B. licheniformis* ATCC14580	4.22	46.19	4,173	21	72	57	116
*B. licheniformis* 9945A	4.38	45.92	4,225	21	72	67	116
*B. licheniformis* WX-02	4.29	46.06	4,512	24	79	62	103

### DNA Methylation Features in *B. licheniformis* DW2

DNA methylation is an essential epigenetic modification that can change the activity of a DNA segment without changing the sequence. It typically represses gene transcription if it locates in a gene promoter (Laird, 2010). In bacteria DNA methylation often acts as a cellular defense system against phage infection that confers a selective advantage to the host bacterium (Sitaraman, 2016). If a foreign DNA is introduced into the cell, it will be degraded by sequence-specific restriction enzymes while the methylated DNA of the bacteria is not recognized by the restriction enzymes. Therefore, DNA methylation can be viewed as a primitive immune system to protect bacteria from bacteriophage attack (Sitaraman, 2016). DNA methylation transfers methyl groups from adenosylmethionine to adenine or cytosine by DNA methyltransferase to form 6-methyladenine (6mA), 4-methyl cell (4-Methylcytosine, 4mC) and 5-Methylcytosine (5mC), of which 6mA and 4mC are mainly found in prokaryotes (Kumar et al., 1994; Sánchezromero et al., 2015). PacBio SMRT technology has been used to simultaneously detect DNA methylation modification during single-molecule genome sequencing (Flusberg et al., 2010). In order to characterize the methylation of *B. licheniformis* DW2, using the SMRT Analysis Modification and Motif detection, we identified 2,939 6 mA, 2,784 4 mC and 88,319 unspecific “modified bases” where the type of modification was not recognized by the software.

We then detected candidate methylation motifs using a sliding window of 5 kb, and identified two dominant motifs (GCANNNNNNNNRTRTC and GAYAYNNNNNNNNTGC), both of them were recognized by N-6 adenine-specific methyltransferases. **Figure 2B** shows the distribution of two motifs in *B. licheniformis* DW2 genome, approximately 40% of 6 mA bases are clustered into the two motifs. However, there is no consensus motif for 4mC-methylated bases or other unspecific modified bases. We aligned the two motifs to a comprehensive restriction-modification (RM) system database REBASE (Roberts et al., 2015) to make sure whether the two motifs have been identified in other species, and found that both motifs could not match existing recognition sequences of the restriction systems.

### Prediction and Analysis of Operons in *B. licheniformis* DW2

We predicted 1021 operons based on RNA-seq data of *B. licheniformis* DW2 using software DOOR. Among these operons, 446 (43.7%) of them are composed of two genes and 19 of them are composed of more than 10 genes. **Figure 2C** shows the operon acoABCL associated with acetoin catabolism, where *acoR* is a transcriptional regulator, *acoL* is dihydrolipoamide dehydrogenase, and *acoABC* is a gene related to acetoin dehydrogenase (Ali et al., 2001). Many microorganisms can convert carbohydrates into acetone during glycolysis to avoid over-acidification. As shown in the figure, these genes had high expression at 22 and 25 h in DW2 strain, while they were almost not expressed at 14 h in DW2 and all the three time points in ΔAbrB strain. These results indicate that AbrB may have a great effect on the expression of this operon, and this effect is time-specific, which has a significant effect in stationary phase and no obvious effect in logarithmic phase.

**Figure 2D** shows the stage V sporulation protein. The *spoVA* operon of *B. subtilis* is expressed only in the developing spore during sporulation and at least five *SpoVA* proteins, *SpoVAA, -B, -C, -D*, and *-Eb*, are necessary for normal *B. subtilis* spore formation (Perez-Valdespino et al., 2014). Many genes of ΔAbrB strain showed much higher expression than DW2 strain, which fitted the basic fact that AbrB was a global regulatory gene and had inhibition effect on many genes. The expression level significantly increased when AbrB gene was knocked out. The ΔAbrB genes at 14 h were highly expressed compared with that at 22 and 25 h. In general, *B. licheniformis* would not sporulate during logarithmic growth phase. As for *spoVAC* and *spoVAB*, we observed that the expression at 22 and 25 h were significantly lower than those at 14 h, which indicated they were activated during logarithmic growth phase. These results suggest that knockout of AbrB is favorable for sporulation. Then the knockout of AbrB may be able to directly relieve the inhibition of AbrB on other genes related to spore synthesis, thereby increasing the transcription level of spore-related genes and promote the synthesis of spores.

### Differentially Expressed Genes (DEGs) of Wild and ΔAbrB *B. licheniformis* DW2 Strains under Different Conditions

**FIGURE 3:**
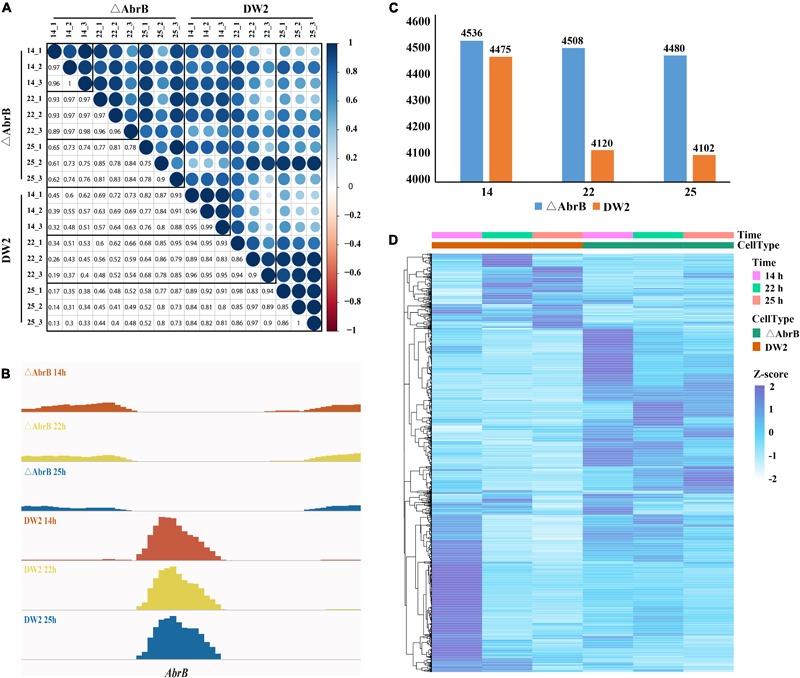
Gene expression of wild *B. licheniformis* DW2 and AbrB-knockout strains. **(A)** The correlation of three biological replicates in three time points of wild and AbrB-knockout strains. **(B)** The AbrB gene was knocked out and not expressed in AbrB-knockout strain. **(C)** The number of expressed genes in three different time points of wild and AbrB-knockout strains. **(D)** The different gene expression pattern between wild and AbrB-knockout strains.

In the experiment of RNA isolation and preparation, we performed three biological replicates. Consequently, we obtained RNA-seq data at three time points from two strains and three biological replicates, 18 sets of RNA-seq data in total. It is important to examine the correlation of the three biological replicates. We calculated the Spearman coefficient of correlation among the data sets. The results in **Figure 3A** show that the RPKM of biological replicates are highly correlated. The AbrB gene has been completely knocked out according to the expression levels (**Figure 3B**).

We screened the expressed genes of wild-type strain and ΔAbrB strain at different time points with the threshold of RPKM > 1, and the number of expressed genes in different time points were in the range of 4,102∼4,536 (**Figure 3C**). DEGs were obtained using DEseq package between the two data sets of both strains and three growth phases (**Table 3**). As shown in **Figure 3D**, the number of expressed genes of ΔAbrB increased significantly than that of wild type in all three time points, indicating many inhibited genes expressed after AbrB gene was knocked out. In **Table 3**, more than a few hundred genes were up-regulated and down-regulated between wild type and ΔAbrB strains in all time points, which supported the above conclusion that AbrB can regulate numerous genes directly and indirectly. The number of expressed genes of DW2 wild type at 14 h is greater than that at 22 and 25 h. It is shown that more genes are activated in logarithmic growth phase, while quite a few of them are absent or very lowly expressed in stationary and transitional phases. Both up-regulated and down-regulated genes in ΔAbrB are far fewer than that of wild type, as shown in **Table 3**, may be caused by the inhabitation absence of AbrB. Many regulated genes expressed at all time points after AbrB was knocked out, and no longer were differentially genes. We also note that the number of DEGs correlate with time length. Among three columns either in wild type or ΔAbrB type, the middle column represents the DEGs numbers between 25 and 14 h, which are significantly greater the others. On the contrary, the DEGs between 22 and 25 h with the shortest time length correspond to the smallest numbers. It suggested that more and more genes begin to transcript or stop transcription and become DEGs with the development process of organism. To gain insight into the differential expression of these DEGs, complete linkage hierarchical cluster analysis based on RPKM values was performed using R. The heat map showed significant differential expression between strains, we identified six broad clusters of genes that exhibited expression changes over time. Genes in cluster found that about 30% of the DEGs in DW2 strains show high expression at logarithmic growth phase (14 h) and low expression at transitional phase (22 h) and stationary phase (25 h).

**Table 3:** The number of DEGs between wild strain and knockout strain.

	ΔAbrB-DW2			ΔAbrB strain			DW2 wild strain		
	14 h	22 h	25 h	14–22 h	14–25 h	22–25 h	14–22 h	14–25 h	22–25 h
Total	1517	1439	1416	575	950	369	1651	1897	710
Up	808	790	827	311	406	115	870	965	291
Down	709	649	589	264	544	254	781	932	419

**FIGURE 4:**
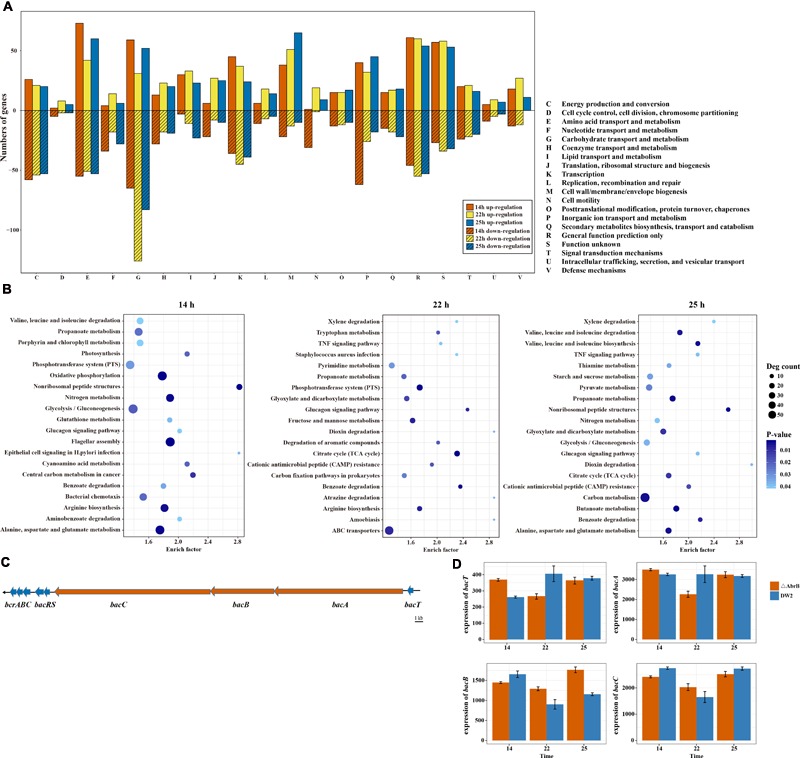
Functional enrichment of DEGs and different expression of bacitracin biosynthesis related genes. **(A)** The functional categories of DEGs in three different time points of wild type and AbrB-knockout strains. **(B)** The top 20 significantly enriched pathways of DEGs in three time points. **(C)** The cluster of bacitracin biosynthesis related genes in *B. licheniformis* DW2. **(D)** The different expression of bacitracin biosynthesis related genes *bacA, bacB, bacC* and *bacT* between wild and AbrB-knockout strains.

To understand the function of these DEGs, they were grouped according to their COG functional categories. As shown in **Figure 4A**, a majority of the functional categories in *B. licheniformis* DW2 contained both induce and repressed genes, indicating that this bacterium needs to balance many biological pathways under different conditions. We found that the pathways had a very unequal distribution, and some of them were significantly affected, such as ‘Carbohydrate transport and metabolism (G),’ ‘Amino Acid transport and metabolism (E),’ ‘Cell wall/membrane/envelope biogenesis (M),’ ‘Energy production and conversion (C)’ and ‘Lipid transport and metabolism (I).’ Notably, almost all DEGs in the functional categories of ‘cell motility (N)’ were down-regulated in logarithmic growth phase (14 h) and up-regulated in transitional phase (22 h) and stationary phase (25 h). Previous observations suggested one of the major functions of AbrB in repressing biofilm formation by repressing signal peptidase *sipW*, and *sipW*-processed protein may have a role in a motility structure in *B. subtilis* (Hamon et al., 2004). We speculated that AbrB repressed some genes related to cell motility in transitional phase (22 h) and stationary phase (25 h).

In addition, we mapped all DEGs to KEGG terms to identify significantly enriched metabolic pathways and got 190 pathways, which contained 1,271 DEGs. To obtain a better visual display of the results, top 20 significantly enriched pathways of each time points were shown in **Figure 4B**. The pathway flagellar assembly was significantly at logarithmic growth phase (14 h), which was consistent with the results of COG function enrichment. We also found that the number of genes in significantly enriched pathways was different among three time points, 14 h is much more than 22 and 25 h. These results also coincide with the variations of DEGs RPKM in ΔAbrB strain.

### The Expression of Bacitracin Biosynthesis Related Genes

Bacitracin, comprised of 12 cyclic polypeptides, is an important non-ribosomal peptide antibiotic. It is produced by several *Bacillus* strains that are usually active against other Gram-positive and some strains of Gram-negative microorganisms, but not against the strain itself. *B. subtilis* C126 was reported as a bacitracin producer (Azevedo et al., 1993). *B. subtilis* 168, which has no bacitracin synthetase, but is more sensitive to bacitracin than *B. licheniformis*, has several homologs of *bcrABC* (Ohki et al., 2003). In previous studies, the bacitracin synthetase gene cluster was clearly described (Konz et al., 1997; Neumüller et al., 2001; Mascher et al., 2003). A thioesterase *bacT* and a non-ribosomal peptide synthetase (NPRS) *bacABC* operon compose the bacitracin synthetase gene cluster in *B. licheniformis*. The *bcrABC* genes of transporter system which are hypothesized to pump out bacitracin from the cells localize downstream of *bacABC*, between the *bacABC* operon and transporter system *bcrABC* is two-component system *bacRS*. Using antiSMASH 4.0.0 tool (Blin et al., 2017), we identified a complete bacitracin synthetase gene cluster in *B. licheniformis* DW2, and all genes of this cluster shows high similarity (identity > 90%) to bacitracin synthetase operon in *B. licheniformis* strain ATCC 10716 (**Figure 4C**). The *bacT* gene is 705 bp and encodes a protein of 235 amino acids. The *bacABC* gene cluster starts from downstream of the *bacT* gene and encoding bacitracin synthetase, it consists of the gene *bacA* (15,771 bp), *bacB* (7,809 bp), and *bacC* (19,080 bp). Gene *bacR* (717 bp) and *bacS* (1,035 bp) encode two-component regulatory system *bacRS*. The *bcrABC* gene cluster locates after 80 bp downstream of the *bacRS* genes, which is composed of *bcrA* (921 bp), *bcrB* (735 bp), and *bcrC* (612 bp) and encoding ABC transporter. These findings provided gene level information of bacitracin biosynthesis for *B. licheniformis* DW2.

Previous study suggested that AbrB might repress the expression of both *bacT* and *bacA* through binding to the promoter regions in *B. licheniformis* (Wang et al., 2017). Although *bacABC* and *bacT* are in the same bacitracin synthetase gene cluster, their transcription levels tremendously vary in both wild type and ΔAbrB (**Figure 4D**). The highest expressed gene *bacA* has about 10-fold RPKM comparing with *bacT*, the lowest expressed one; while *bacB* and *bacC* are between the two. In addition, the genes of bacitracin synthetase cluster did not share an identical differentiated expression manner when AbrB was knocked out, the up/down regulations of *bacT* and *bacABC* did not show an obvious regularity. However, at 22 h their expression level of ΔAbrB were lower than those of 14 and 25 h, which was found in all the above four genes, especially in *bacT* and *bacA*.

Notably, the *bcrABC* were significantly up-regulated in ΔAbrB strain at all three time points in contrast to wild type (Supplementary Figure S3). In previous study, *B. licheniformis* was hypothesized to pump out bacitracin for self-resistance by *bcrABC* (Neumüller et al., 2001). Besides, experiments showed that ΔAbrB strains would increase bacitracin yield (Supplementary Figure S2; Wang et al., 2017). Our findings provided evidence for bacitracin up-regulation and transport, which was accordance with the above two studies and linked them together. Comparing with wild type, AbrB-knockout strains secreted more bacitracin, thus they had to increase resistance to the extra bacitracin in order to avoid self-intoxication. The up-regulation of *bcrABC* enabled ΔAbrB strain produce more transporters to remove the bacitracin out of the cell. So it is reasonable to view the differential expression of *bcrABC* as a response to the change of bacitracin production.

## Conclusion

In this study, we successfully assemble the complete genome of *B. licheniformis* DW2 using PacBio SMRT sequencing system and *de novo* assembly based on the HGAP method. The genomic information obtained in this study would help subsequent comparative genomic analysis with other *B. licheniformis* strains. Meanwhile, we conducted comprehensive analyses of *B. licheniformis* DW2 by ssRNA-seq in wild and ΔAbrB strains. The ssRNA-seq data enabled us to comparatively analyze DEGs between different strains and growth phases and provided us information on bacitracin synthesis. In previous studies, *B. licheniformis* was considered to transport bacitracin for self-resistance by *bcrABC* (Neumüller et al., 2001). The global transcription regulator AbrB represses the transcription of the bacitracin synthase operon by directly binding to the bacitracin synthase operon promoter region (Wang et al., 2017). In our study, we identified a complete bacitracin synthetase gene cluster in the genome including the location and length of each gene. The expressions of *bcrABC* were significantly upregulated in ΔAbrB strain. These findings provided evidence for bacitracin up-regulation and removing out of the cell, which were consistent with the above two studies and linked them together. This study would be helpful for improving the industrial process of bacitracin production through AbrB-knockout and further understanding of bacitracin transport in *B. licheniformis*.

## Accession Numbers

The genomic and transcriptomic sequencing data of *B. licheniformis* DW2 generated in this study have been submitted to BIG Data Center (http://bigd.big.ac.cn) under accession numbers GWHAAAE00000000 and CRA000557.

## Author Contributions

L-LC and J-XG conceived the study. C-CS, JG, and J-MS analyzed the data. S-WC and DW performed the experiments. C-CS, L-LC, and J-XG wrote the paper.

## Conflict of Interest Statement

The authors declare that the research was conducted in the absence of any commercial or financial relationships that could be construed as a potential conflict of interest.

## Footnotes

1 https://cge.cbs.dtu.dk/services/Restriction-ModificationFinder-1.0/

2 http://www.expasy.ch/sprot

3 http://www.ncbi.nlm.nih.gov
