# Supplementary material for: Analyzing AbrB-Knockout Effects through Genome and Transcriptome Sequencing of Bacillus licheniformis DW2
Source: Front Microbiol. 2018 Feb 26;9:307. doi: 10.3389/fmicb.2018.00307 (PMC5863516; doi:10.3389/fmicb.2018.00307)
Supplement: Supplementary file 1 [file Image_1.PDF]

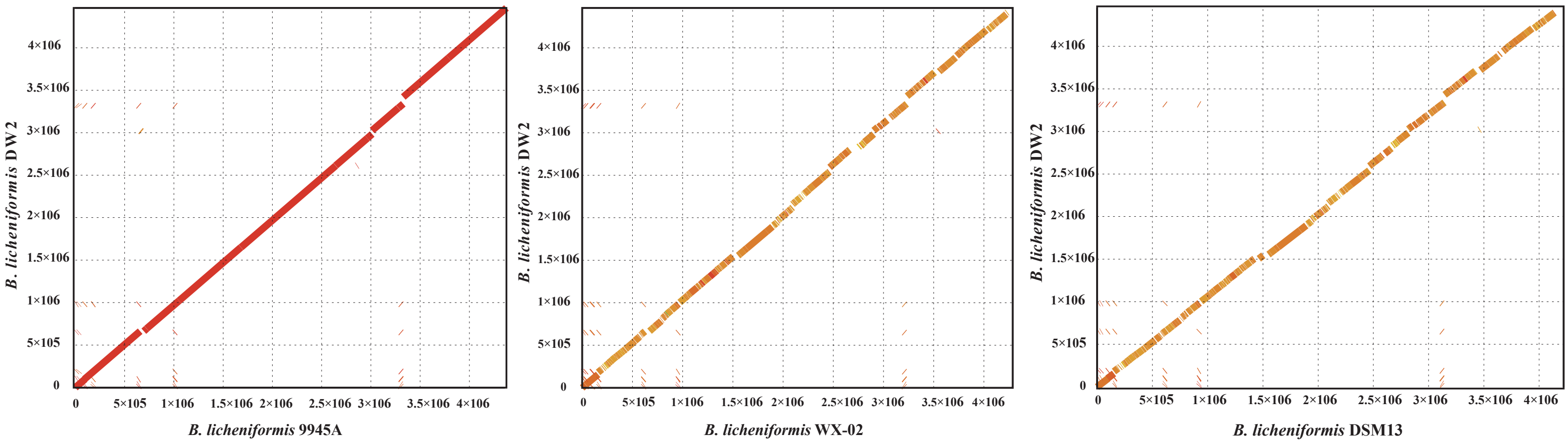

FIGURE S1 | The B. licheniformis DW2 genome is collinear with B. licheniformis 9945A, B. licheniformis WX-02 and B. licheniformis DSM13, respectively.
