# Supplementary figures and images for: Analyzing AbrB-Knockout Effects through Genome and Transcriptome Sequencing of Bacillus licheniformis DW2
Source: Front Microbiol. 2018 Feb 26;9:307. doi: 10.3389/fmicb.2018.00307 (PMC5863516; doi:10.3389/fmicb.2018.00307)

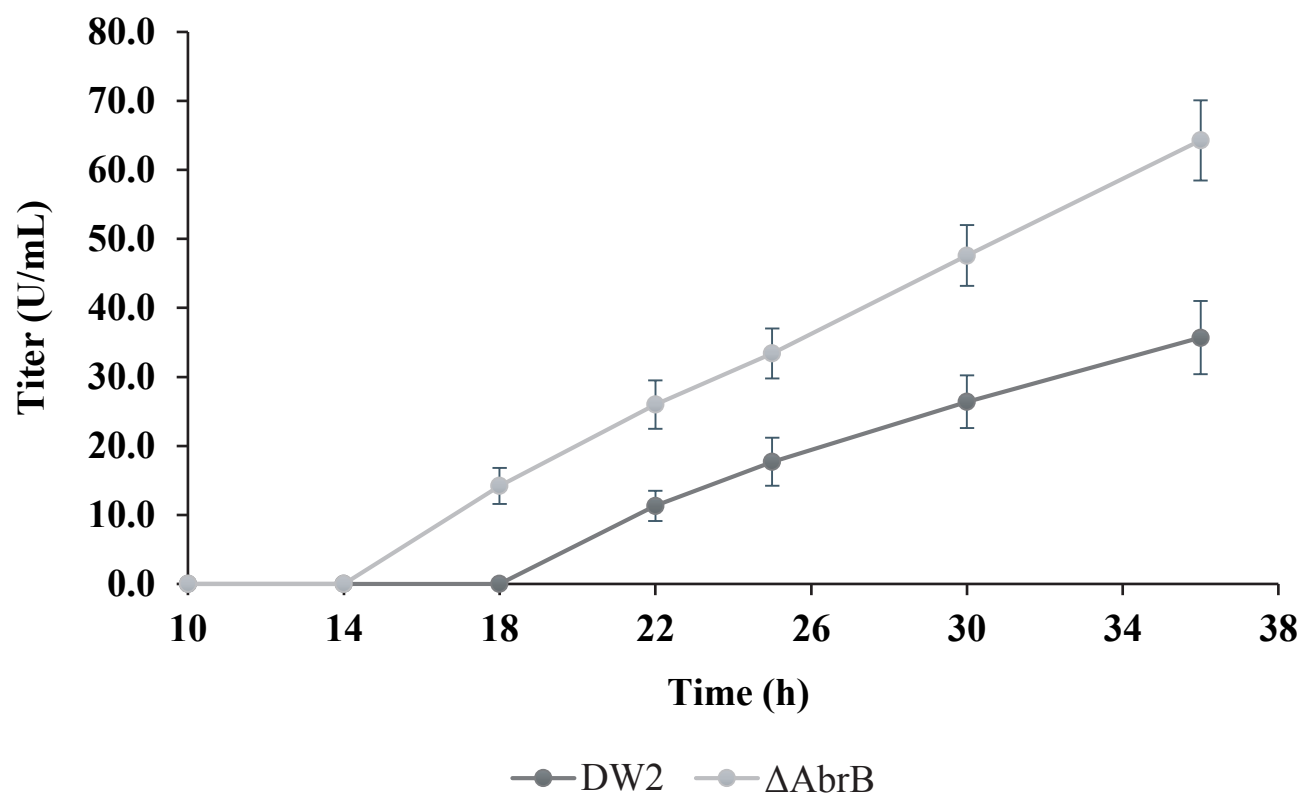

**FIGURE S2 | The comparison of bacitracin production between wild-type and  $\Delta$ AbrB.**

Supplement: Supplementary file 2 [file Image_2.PDF]

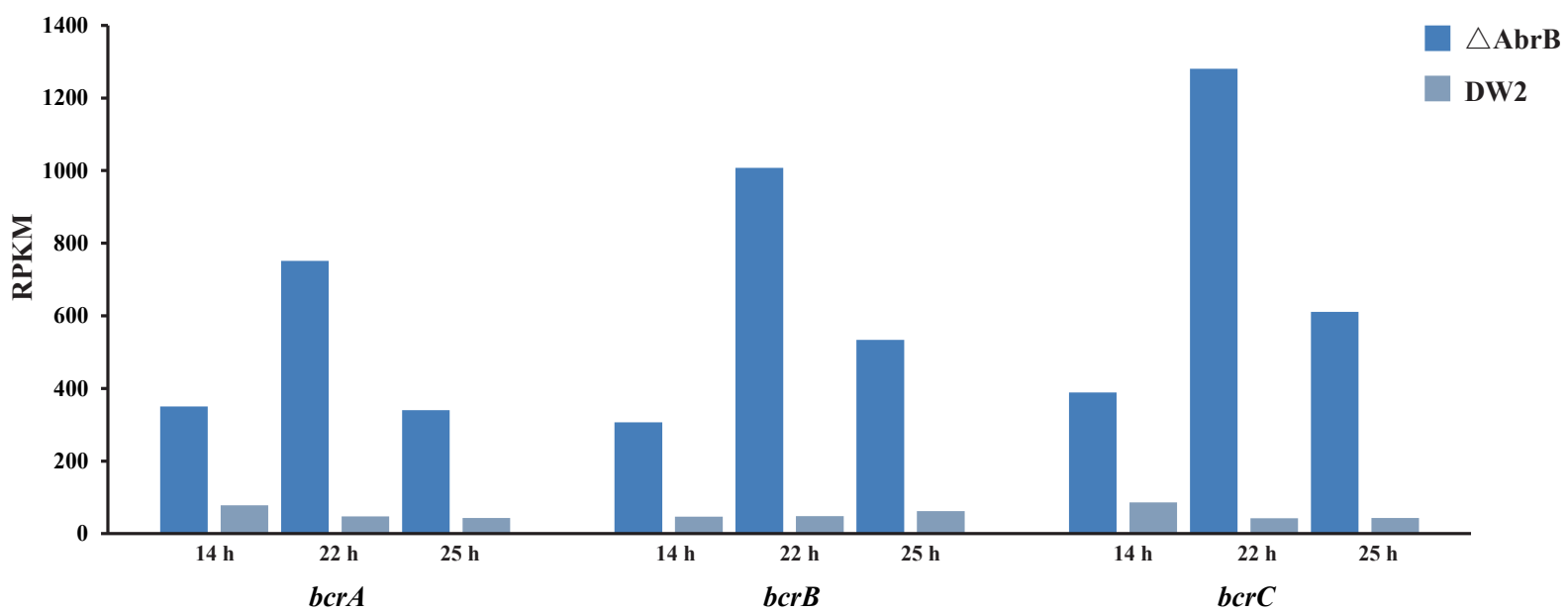

**FIGURE S3 | The comparison of *bcrABC* expression level between wild-type and  $\Delta$ AbrB.**

Supplement: Supplementary file 3 [file Image_3.PDF]
